# Polyneuropathy in hereditary and wildtype transthyretin amyloidosis, comparison of key clinical features and red flags

**DOI:** 10.1038/s41598-025-21745-5

**Published:** 2025-10-07

**Authors:** Janna M. Siemer, Lea Grote-Levi, Anja Hänselmann, Mieke L. Sassmann, Sandra Nay, Dominica Ratuszny, Sonja Körner, Tabea Seeliger, Martin W. Hümmert, Maike F. Dohrn, André Huss, Hayrettin Tumani, Vega Gödecke, Michael Heuser, Johann Bauersachs, Udo Bavendiek, Thomas Skripuletz, Stefan Gingele

**Affiliations:** 1 Department of Neurology, Hannover Medical School, Carl-Neuberg-Str. 1, 30625 Hannover, Germany; 2 Department of Cardiology and Angiology, Hannover Medical School, Hannover, Germany; 3 Amyloidosis Center Lower Saxony, Hannover Medical School, Hannover, Germany; 4 Department of Neurology and Neuromuscular Center, Medical Faculty, RWTH Aachen University, Aachen, Germany; 5 Department of Neurology, University Hospital Ulm, Ulm, Germany; 6 Department of Nephrology, Hannover Medical School, Hannover, Germany; 7 Department of Hematology, Hemostasis, Oncology, and Stem Cell Transplantation, Hannover Medical School, Hannover, Germany

**Keywords:** ATTRv amyloidosis, ATTRwt amyloidosis, Polyneuropathy, Red flags, Serum neurofilament light chain, Neurology, Peripheral neuropathies

## Abstract

Transthyretin (TTR) amyloidosis manifests in two distinct forms: hereditary (ATTRv) and wild-type transthyretin amyloidosis (ATTRwt). Despite being one of the commonest manifestations in ATTRv amyloidosis, the presence of polyneuropathy has long been underestimated in ATTRwt patients. This prospective study enrolled 72 patients with ATTRv (*n* = 11) and ATTRwt (*n* = 61) amyloidosis. Our standardized protocol included a detailed patient history, clinical and electrophysiological examinations, assessment of unrelated neuropathy risk factors and predefined red flags for ATTRv amyloidosis, as well as serum neurofilament light chain concentrations (NfL). We found signs of polyneuropathy in all ATTRv patients and a vast majority of ATTRwt patients (84%). Predefined red flag symptom clusters were prevalent in both subgroups, indicating significant overlap, however gastrointestinal symptoms were more frequent in ATTRv amyloidosis (*p* = 0.008), while carpal tunnel syndrome was less common (*p* = 0.015) compared to ATTRwt amyloidosis. The groups differed in severity of polyneuropathy, with ATTRv patients demonstrating more pronounced subjective limitations, greater clinical disability, marked nerve conduction abnormalities, and higher serum NfL concentrations (*p* = 0.011). Our findings underscore a high prevalence of polyneuropathy in patients with transthyretin amyloidosis, irrespective of its origin. Differences in the severity of polyneuropathy as well as in red flags indicate different underlying mechanisms of damage.

## Introduction

Transthyretin amyloidosis (ATTR amyloidosis) is a progressive, multisystemic disease characterized by the accumulation of amyloid fibrils in various tissues and organs^1,2^. It manifests in two forms: hereditary transthyretin amyloidosis (ATTRv), which is inherited in an autosomal dominant manner, and acquired wild-type transthyretin amyloidosis (ATTRwt)^3^. ATTRv amyloidosis develops on the basis of a pathogenic variant in the transthyretin (*TTR*) gene, leading to transthyretin tetramer dissociation, misfolding of transthyretin monomers and formation of amyloid fibrils^4^. Currently, over 130 pathogenic variants of the *TTR* gene have been described^5^. Historically, ATTRv amyloidosis has been associated primarily with neurologic manifestations in the form of rapidly progressive sensorimotor and autonomic polyneuropathy in addition to cardiomyopathy, gastrointestinal, renal, or ocular disorders^6^. However, clinical manifestations vary considerably depending on the respective variant and other factors such as occurrence in an endemic vs. a non-endemic region^3,7^. Therefore, “red flag” symptom clusters with findings considered typical were defined to facilitate the diagnosis of ATTRv amyloidosis^8^.

In contrast to ATTRv amyloidosis, the pathophysiology of ATTRwt amyloidosis has been far less understood. ATTRwt amyloidosis typically occurs in the elderly population with a strong male-dominated gender distribution^9^. Since cardiomyopathy is often the most prominent symptom in ATTRwt amyloidosis, concomitant neuropathy has long been underdiagnosed^10^. However, recent studies have drawn attention to a higher-than-expected prevalence of neuropathy in ATTRwt patients and further characterized peripheral neuropathy in ATTRwt amyloidosis as predominantly mild^10,11^. Nevertheless, there are only limited data on the characterization of polyneuropathy in ATTRwt patients and comprehensive comparison of the features of neuropathy in ATTRv and ATTRwt amyloidosis.

Therefore, in this study we aimed to identify the prevalence as well as clinical and electrophysiological features of polyneuropathy in a mixed cohort of patients with hereditary and acquired transthyretin amyloidosis at first presentation at an interdisciplinary amyloidosis center in a non-endemic area.

## Materials and methods

### Patients

Between September 2019 and June 2022, a total of 72 patients with a diagnosis of transthyretin amyloidosis—either hereditary (ATTRv) or wild-type (ATTRwt)—were enrolled in this study at their first neurological presentation at the Amyloidosis Center Lower Saxony at Hannover Medical School. All patients were seen on an interdisciplinary basis in the Department of Cardiology and the Department of Neurology. Prior to definite diagnosis, all patients were genetically tested for the presence of a pathogenic variant in the transthyretin gene. The diagnosis of ATTR amyloidosis was based on the detection of a pathogenic variant combined with typical clinical findings of amyloidosis, histopathological detection of transthyretin amyloid in different tissues and/or positive cardiac uptake in 99^m^ Tc scintigraphy after laboratory exclusion of monoclonal light chain disease according to the guidelines of the German Cardiac Society (DGK)^12^. All patients provided written informed consent before inclusion in this prospective observational study. This investigation was approved by the Ethics Committee of Hannover Medical School (No. 9741) and all research was performed in accordance with the relevant guidelines and regulations.

### Procedures

We assessed demographic data such as age and sex. In patients with ATTRv amyloidosis the specific amyloidogenic variant was noted. Patients underwent a detailed and standardized neurological assessment including patient history with emphasis on amyloidosis or neuropathy-associated diseases, clinical examination, laboratory tests investigating different causes of neuropathies, and nerve conduction studies (NCS). Additionally, we measured serum concentrations of neurofilament light chain (NfL).

### Neurological examinations

All patients were interviewed in detail about neurological symptoms and diagnoses and underwent a detailed and standardized neurological examination by a senior neurologist. The diagnosis of polyneuropathy (PNP) was based on three components of (a) subjective neuropathic symptoms, (b) typical clinical signs of PNP and (c) electrophysiological findings in accordance with PNP. First, typical PNP symptoms, i.e. distal-symmetrical sensory disturbances, gait instability and loss of muscle strength were asked. As part of the standardized neurological examination, clinical signs typical of PNP were identified and categorized: distal-symmetric an-/ hypoesthesia, pallan-/hypoesthesia, paresis, lack of ≥ 2 tendon reflexes, ataxia/instability in gait tests and positive/not possible Romberg test. Patients were considered to show clinical signs of polyneuropathy if at least 2 of these clinical categories were met. Additionally, nerve conduction studies (NCS) were carried out to support or exclude the diagnosis of PNP. Patients were diagnosed with polyneuropathy if at least 2 of the 3 categories were fulfilled, except when nerve conduction studies were normal, the diagnosis of PNP was excluded. Disabilities and limitations in daily life were additionally evaluated by questionnaires. To evaluate activity and limitations on social participation, the Inflammatory Rasch-built Overall Disability Scale (I-RODS) was performed and raw values (0–48) were calculated, with lower values indicating greater limitations^13^. The Inflammatory Neuropathy Cause and Treatment disability score (INCAT) (ranging from 0 to 10, with higher values displaying greater disability) was used to assess polyneuropathy-associated disability^14^. Muscle strength was quantified by the Medical Research Council (MRC) Sum Score for eight muscle groups (shoulder abduction, elbow flexion, wrist extension, index finger abduction, hip flexion, knee extension, foot dorsiflexion, and great toe dorsiflexion; range of total score: 0–80)^15^. Polyneuropathy severity was further evaluated with a focus on walking ability using the familial amyloid polyneuropathy (FAP) and the polyneuropathy disability (PND) scores. The FAP score contains stages 0 to 3: 0 = asymptomatic, 1 = symptomatic but ambulatory without aid, 2 = ambulatory with aid, 3 = wheelchair-bound or bedridden. The PND score includes a similar classification: 0 = no impairment, I = sensory disturbances, preserved walking ability, II = impaired walking capability but ambulatory without aid, IIIa = walking with one walking aid, IIIb = walking with two walking aids, IV = wheelchair-bound or bedridden^16,17^. In addition, gait function was assessed using the timed 100-meter walk test and the mean of two runs of the timed 25-foot walk test^18^.

### Routine laboratory testing

As part of the clinical routine diagnostics, we conducted a comprehensive laboratory testing for the differential diagnosis of polyneuropathy. This included HbA1c, vitamin B12, folic acid, testing for liver, kidney, and thyroid function, electrolytes, differential white blood cell count, immunofixation, serum free light chains, antinuclear antibodies (ANA), and extractable nuclear antigen antibodies (ENA).

### Nerve conduction studies

Nerve conduction studies (NCS) were conducted in eleven (100%) ATTRv patients and 43 (69%) ATTRwt patients. The measurements were taken unilaterally on the peroneal nerve, tibial nerve, sural nerve, and median nerve and/or ulnar nerve. For all motor nerves composite muscle action potential (CMAP), distal motor latency (DML), motor conduction velocity (MCV), and F-wave latency (FWL) were acquired; sensory conduction velocity (SCV) and sensory nerve action potential (SNAP) were determined for the sensory nerves. The amplitude of the evoked CMAP was determined by base to peak measurement. NCS were performed via surface electrodes and applying standard procedures using the Nicolet EDX^®^ EMG/ NCS/ EP/ IOM System by Natus^19,20^. Results were considered consistent with large-fiber polyneuropathy if ≥ 2 nerves showed abnormal findings in accordance with polyneuropathy without evidence of other causes such as entrapment neuropathy. An axonal damage pattern was assumed if ≥ 2 nerves showed CMAP or SNAP below the lower limit of normal according to in-house standards (see Table 4 for NCS reference values). Isolated reduction of sural SNAP was not sufficient to indicate axonal damage due to age-associated amplitude reduction^21^. A demyelinating component of nerve damage was assumed if ≥ 2 nerves showed prolonged DML, reduced MCV or SCV and/or prolonged FWL which was not explainable by axonal damage.

### Neurofilament light chain measurement

Neurofilament light chain (NfL) in serum as a marker for neuro-axonal damage was detected by the ELLA microfluidic system and the respective assay (Bio-Techne, Minneapolis, USA). Measurements were performed according to the manufacturer‘s instructions, i.e. serum samples were diluted 1:2 und analyzed in technical triplicates with an acceptance threshold of 10% coefficients of variation. NfL levels were considered elevated if they were above the age-specific 95th percentile according to reference values of the laboratory for CSF diagnostics and clinical neurochemistry of University Hospital Ulm.

### Survey of red flag symptoms

Previously established “red flag” symptom clusters for ATTRv amyloidosis were evaluated in ATTRv and ATTRwt amyloidosis patients^8^. Predefined red flags included the presence of polyneuropathy (assessment was based on current neurological diagnostics), a positive family history of polyneuropathy, autonomic dysfunction (evaluation via questionnaire regarding erectile dysfunction, excessive or reduced sweating, diarrhea, constipation or both alternating, and orthostatic dysregulation), gastrointestinal (GI) complaints, and history of unexplained weight loss, cardiac involvement (evaluation by current cadiologic diagnostics), carpal tunnel syndrome (including previous and current diagnoses), renal abnormalities (defined as eGFR < 60 ml/min and/or albuminuria > 0.03 g/g creatinine)^22–24^, and vitreous opacities (positive history of vitreous opacities due to amyloid)^8^. Red flag symptoms were evaluated at the initial appointment at the Department of Neurology. Diagnosis of carpal tunnel syndrome was made if it was (i) prediagnosed (and treated) in the past, and/or (ii) classic symptomatology described by the patient, and/or (iii) typical clinical signs (e.g. thenar atrophy) present in combination with typical electrophysiological findings (especially a predominant sensory involvement and prolonged DML)^25,26^.

### Statistics

Statistical analyses were performed using GraphPad Prism 5.0 (GraphPad Prism Software, San Diego, CA, USA). Normal distribution was tested by D’Agostino-Pearson omnibus K2 test or in case of low case number by Kolmogorov-Smirnov test. Results were demonstrated as median with 25–75% interquartile range (IQR) or mean with standard deviation, if appropriate. Statistical analysis was performed by using two-tailed t-test or Mann-Whitney-U test when appropriate. Results were considered statistically significant at *p* ≤ 0.05. In detail, significance was classified for different *p*-values (*p* ≤ 0.05(*), *p* ≤ 0.01(**)).

## Results

### Baseline characteristics

In this study, we enrolled 72 patients diagnosed with transthyretin amyloidosis. Of these, 11 patients had ATTRv amyloidosis and 61 had ATTRwt amyloidosis. The median age was significantly higher in patients with ATTRwt amyloidosis at 78 years (IQR: 74–80) compared to 74 years (IQR: 62–78) in those with ATTRv amyloidosis (*p* = 0.032) (Table 1). Patients with ATTRwt amyloidosis showed a higher male predominance (90% male) compared to ATTRv amyloidosis patients (64% male) (*p* = 0.040). Among the ATTRv amyloidosis patients, nine individuals were diagnosed with the p.*Val50Met* amyloidogenic variant and two with *non-*p.*Val50Met* variants: p.*Cys30Arg* and p.*Ile104Thr*. One of the nine p.*Val50Met* variant carriers was from an endemic area. Concomitant diabetes mellitus was present in 18% (*n* = 2/11) of ATTRv and in 16% (*n* = 10/61) of ATTRwt amyloidosis patients, respectively. Of note, diabetic neuropathy represented the most important confounding factor in the analysis. Before the current presentation at the Department of Neurology, 73% (*n* = 8/11) of patients with ATTRv amyloidosis had been previously diagnosed with polyneuropathy, in sharp contrast to only 3% (*n* = 2/61) of those with ATTRwt amyloidosis (*p* = 0.001). Interestingly, CTS was diagnosed more frequently prior to the main disease diagnosis in the ATTRwt group than in the ATTRv group (*p* = 0.047) (Table 1).

**Table 1:** Patient baseline characteristics.

	ATTRv (*n* = 11)	ATTRwt (*n* = 61)	*p*
Age (years), median (IQR)	74 (62–78)	78 (74–80)	**0.032**
Sex, n (%)			
Female	4 (36)	6 (10)	**0.040**
Male	7 (64)	55 (90)	
Mutation, n (%)			
*p.Val50Met* variant	9 (82)	n.a.	
*Non-p.Val50Met* variant	2 (18)	n.a.	
Previous diagnosis of PNP, n (%)	8 (73)	2 (3)	**0.001**
Competing causes of neuropathy, n (%)			
Diabetes	2 (18)	10 (16)	1.0
Monoclonal gammopathy	0 (0)	4 (7)	0.615
Systemic immune-mediated disease (e.g. vasculitis, connective tissue disease)	1 (9)	4 (7)	1.0
Thyroid dysfunction	1 (9)	1 (2)	0.284
Toxic (e.g. alcohol abuse)	0 (0)	1 (2)	1.0
Infectious (e.g. hepatitis B, borreliosis)	0 (0)	4 (7)	0.615
Vitamin B12 deficiency	0 (0)	1 (2)	1.0
Folic acid deficiency	0 (0)	0 (0)	1.0
Previous diagnosis of CTS, n (%)			
Total	2 (18)	33 (54)	**0.047**
One side	0 (0)	3 (5)	1.0
Both sides	2 (18)	30 (49)	0.097

Abbreviations: IQR: interquartile range; PNP: polyneuropathy; CTS: carpal tunnel syndrome. Results were considered statistically significant at p ≤ 0.05.

### Clinical neurological assessments

After their first presentation at the Department of Neurology, all patients with ATTRv amyloidosis were diagnosed with polyneuropathy, as noted in Table 2. Remarkably, a considerable proportion (84%; *n* = 51/61) of patients with ATTRwt amyloidosis was also diagnosed with polyneuropathy. Among these patients, 36% in the ATTRv and 33% ATTRwt group were diagnosed with at least one other potential cause of polyneuropathy, leaving 64% of ATTRv patients and 67% of ATTRwt patients without any other identifiable etiology of polyneuropathy.

**Table 2:** Symptoms and clinical findings in ATTRv and ATTRwt amyloidosis patients.

	ATTRv *n*/11 (%)	ATTRwt *n*/61 (%)	*p*
PNP Diagnosis, n (%)	11 (100)	51 (84)	0.208
Numbness or par-/dysesthesia perceived by patients	8 (73)	26 (43)	0.101
Hypoesthesia in examination	6 (55)	13 (21)	**0.031**
Pallhypoesthesia in examination	11 (100)	52 (85)	0.338
Reduction of muscle strength noticed by patients	3 (27)	5 (8)	0.098
Paresis in examination	6 (55)	14 (23)	0.061
Lack of ≥ 2 tendon reflexes	9 (82)	33 (54)	0.107
Gait insecurity reported by patients	6 (55)	19 (31)	0.173
Ataxia/unsteadiness in gait tests	9 (82)	44 (72)	0.716
Positive/ unfeasible Romberg test	7 (64)	35 (57)	0.753
Newly diagnosed CTS	1 (9)	9 (15)	0.698
Previously diagnosed CTS	2 (18)	33 (54)	**0.047**
Total CTS diagnosis (pre- and newly diagnosed)	3 (27)	42 (69)	**0.015**

Abbreviations: PNP: polyneuropathy; CTS: carpal tunnel syndrome. Results were considered statistically significant at p ≤ 0.05.

Numbness and sensory discomfort were the most commonly reported symptoms, affecting 73% (*n* = 8/11) of ATTRv patients and 43% (*n* = 26/61) of ATTRwt patients followed by reported gait disturbance, affecting 55% (*n* = 6/11) of ATTRv patients and 31% (*n* = 19/61) of ATTRwt patients.

Clinical neurological examinations revealed a higher incidence of polyneuropathy findings than that of the symptoms reported by patients in both groups. For instance, muscle weakness was found in 55% (*n* = 6/11) of ATTRv patients and 23% (*n* = 14/61) of ATTRwt patients during examination, although only 27% (*n* = 3/11) of ATTRv and 8% (*n* = 5/61) of ATTRwt patients had reported a subjective loss of muscle strength. Similar discrepancies were observed with regards to sensory symptoms and gait disturbance (Table 2).

Evaluation of disabilitiy using different clinical scores showed a more severe neurological impairment in patients with ATTRv compared to those with ATTRwt amyloidosis. Neurological assessments using the INCAT, FAP, and PND scores demonstrated more severe clinical disability in ATTRv patients, reflected by a greater percentage of patients displaying an INCAT score of ≥ 4 (ATTRv: 45% vs. ATTRwt 16%, *p* = 0.044), FAP stage of 3 (ATTRv: 18% vs. ATTRwt 0%, *p* = 0.022) and PND score of IV (ATTRv: 18% vs. ATTRwt 0%, *p* = 0.022). Notably, 62% (*n* = 38/61) of ATTRwt patients fulfilled the criteria for a FAP score of 1 and 21% (*n* = 13/61) for a FAP score of 2, underscoring the neurological impairment in ATTRwt patients (Table 3). Furthermore, overall muscle strength, measured by the MRC Sum Score, was significantly lower in ATTRv patients with a median score of 77 (IQR: 57–80), compared to ATTRwt patients, with a median score of 80 (IQR: 79–80) (*p* = 0.007). Despite the observed trend towards more severe limitations in ATTRv patients, no significant differences were found in the RODS score, the timed 100-meter walking test, or the timed 25-foot walking test in the two groups (Table 3).

**Table 3:** Neurological scores for graduation of severity of polyneuropathy abbreviations: INCAT: inflammatory neuropathy cause and treatment Score; IQR: interquartile range; FAP: Familial amyloid polyneuropathy; PND score: polyneuropathy disability Score; RODS: Rasch-built Overall disability scale Score; MRC: medical research Council sum Score. Results were considered statistically significant at p ≤ 0.05.

	ATTRv (*n* = 11)	ATTRwt (*n* = 61)	*p*
INCAT, median (IQR)	2 (0–6)	1 (0–3)	0.129
INCAT < 4, *n* (%)	6 (55)	51 (84)	**0.044**
INCAT ≥ 4, *n* (%)	5 (45)	10 (16)	**0.044**
FAP stage, n (%)			
FAP stage 0	0 (0)	10 (16)	0.208
FAP stage 1	6 (55)	38 (62)	0.740
FAP stage 2	3 (27)	13 (21)	1.0
FAP stage 3	2 (18)	0 (0)	**0.022**
PND score, n (%)			
PND score 0	0 (0)	10 (16)	0.208
PND score I	4 (36)	23 (38)	1.0
PND score II	2 (18)	15 (25)	0.726
PND score IIIa	0 (0)	9 (15)	0.338
PND score IIIb	3 (27)	4 (7)	0.066
PND score IV	2 (18)	0 (0)	**0.022**
RODS, median (IQR)	33 (18–48)	43 (37–45)	0.363
MRC sum score, median (IQR)	77 (57–80)	80 (79–80)	**0.007**
Timed 100 m walking test (seconds), median (IQR)	80 (54–112)	76 (59–86)	0.876
Timed 25ft walking test (seconds), median (IQR)	6.6 (4.2–7.8)	5.7 (4.5–6.6)	0.772

### Nerve conduction studies

Nerve conduction studies (NCS) revealed symmetric sensorimotor large-fiber polyneuropathy in 82% (*n* = 9/11) of patients with ATTRv amyloidosis. Among these patients, four patients exhibited predominantly an axonal damage pattern, while the other five patients demonstrated a mixed axonal-demyelinating pattern, despite the primary etiology could not be clearly determined. Two patients with ATTRv amyloidosis had normal NCS at the intitial presentation. However, both patients displayed clear clinical signs of polyneuropathy and showed NCS abnormalities during annual follow-up examinations.

Of the ATTRwt patients examined electroneurographically, 81% (*n* = 35/43) displayed signs of large-fiber polyneuropathy. Of these, 32 patients had sensorimotor involvement, and three displayed purely motor nerve fiber involvement. Furthermore, 20 ATTRwt patients showed a pure or predominantly axonal damage pattern, twelve had a mixed pattern, and three exhibited a primarily demyelinating pattern (Table 4). In addition, three ATTRwt patients had abnormal NCS findings only in one nerve, and three patients had typical findings of CTS as the sole finding. Only two ATTRwt patients had completely normal NCS results. The proportion of nerves that could not be electroneurographically evaluated was significantly higher in ATTRv patients compared to ATTRwt patients, reflecting more severe peripheral nerve damage in the ATTRv group, as also indicated by clinical scores (Table 4).

**Table 4:** Nerve conduction studies.

	Derivable nerves			Not derivable nerves		
	ATTRv Mean ± SD	ATTRwt Mean ± SD	*p*	ATTRv [%]	ATTRwt [%]	*p*
Peroneal nerve	*n* = 11	*n* = 43				
DML [≤ 6.5 ms]/ 7–11 cm	5.0 ± 0.7	5.4 ± 1.0	0.370	45%	5%	**0.003**
CMAP [≥ 2 mV]	2.5 ± 2.4	4.0 ± 2.7	0.226	45%	5%	**0.003**
MCV [≥ 40 m/s] (distal)	44.3 ± 6.7	40.2 ± 6.4	0.148	45%	5%	**0.003**
MCV [≥ 40 m/s] (proximal)	49.5 ± 14.4	55.2 ± 14.9	0.388	45%	5%	**0.003**
FWL [≤ 61 ms]	48.4 ± 13.0	55.1 ± 7.2	0.119	56%	14%	**0.039**
Tibial nerve	*n* = 10	*n* = 38				
DML [≤ 8.1 ms]/ 7–11 cm	6.0 ± 2.5	4.8 ± 1.2	0.173	40%	0%	**0.001**
CMAP [≥ 4 mV]	5.6 ± 7.3	5.1 ± 3.2	0.770	40%	0%	**0.001**
MCV [≥ 40 m/s] (distal)	42.8 ± 7.8	41.5 ± 8.5	0.993	40%	0%	**0.001**
FWL [≤ 63 ms]	53.9 ± 6.3	55.1 ± 7.2	0.759	44%	0%	**0.001**
Sural nerve	*n* = 11	*n* = 43				
SCV [≥ 40 m/s]	47.7 ± 6.5	45.4 ± 7.6	0.658	73%	42%	0.095
SNAP [≥ 4 microV]	9.1 ± 2.9	5.0 ± 2.1	**0.015**	73%	42%	0.095
Median nerve	*n* = 10	*n* = 35				
DML [≤ 4.5 ms]/ 7–8 cm	5.0 ± 1.6	5.0 ± 1.1	0.621	10%	0%	0.222
CMAP [≥ 7 mV]	4.9 ± 3.4	6.8 ± 2.6	0.076	10%	0%	0.222
MCV [≥ 45 m/s] (distal)	45.8 ± 10.0	50.2 ± 5.6	0.239	10%	0%	0.222
FWL [≤ 30 ms]	45.8 ± 10.0	39.8 ± 47.6	0.633	33%	0%	**0.006**
SCV [≥ 45 m/s]	46.7 ± 5.1	38.2 ± 8.3	**0.035**	70%	28%	**0.025**
SNAP [≥ 5 mikroV]	8.5 ± 7.9	6.3 ± 3.3	0.965	70%	28%	**0.025**
Ulnar nerve	*n* = 8	*n* = 21				
DML [≤ 3.3 ms] / 7–8 cm	4.1 ± 1.8	3.1 ± 0.5	0.115	0%	0%	1.0
CMAP [≥ 4 mV]	5.8 ± 4.4	9.4 ± 1.8	**0.004**	0%	0%	1.0
MCV [≥ 45 m/s] (above sulcus)	43.9 ± 15.3	52.0 ± 8.9	0.085	0%	0%	1.0
MCV [≥ 45 m/s] (below sulcus)	50.8 ± 12.8	48.9 ± 12.1	0.713	0%	0%	1.0
FWL [≤ 31 ms]	31.7 ± 3.4	30.0 ± 3.6	0.245	13%	0%	0.276
SCV [≥ 45 m/s]	52.3 ± 6.5	53.0 ± 9.9	0.843	63%	29%	0.198
SNAP [≥ 4 mikroV]	3.0 ± 0.9	5.1 ± 1.9	0.052	63%	29%	0.198

Abbreviations: CMAP: composite muscle action potential; DML: distal motor latency; MCV: motor conduction velocity; FWL: F-wave latency; SCV: sensory conduction velocity; SNAP: sensory nerve action potential; SD: standard deviation. Results were considered statistically significant at p ≤ 0.05.

### Neurofilament light chain measurements

**Fig. 1:**
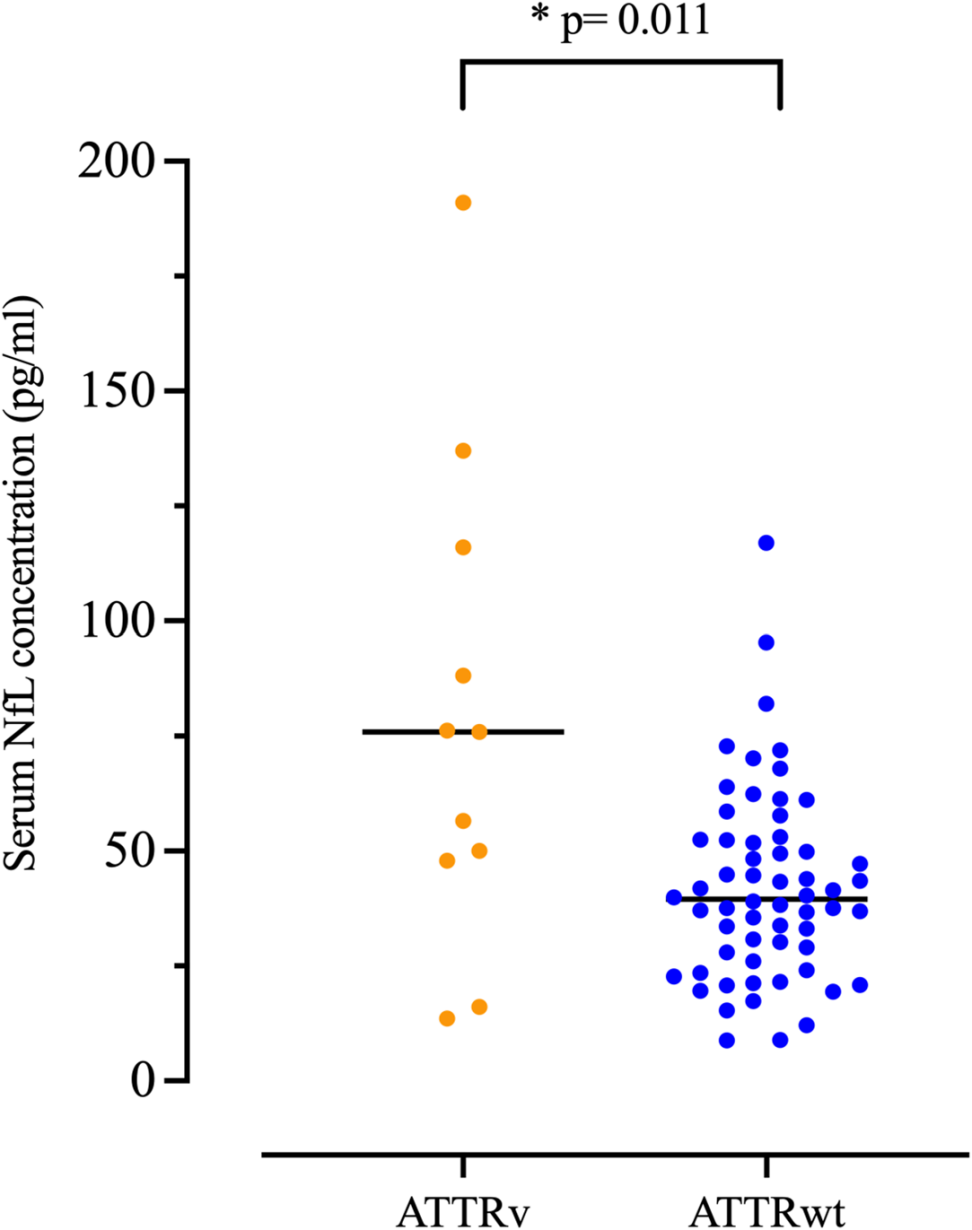
Measurement of serum levels of neurofilament light chain (NfL) in ATTRv (*n* = 11) and ATTRwt (*n* = 60) patients. Dots indicate individual serum NfL values, lines indicate median. Note the elevated serum NfL levels in both subgroups, with significantly higher values in ATTRv patients (*p* = 0.011). Abbreviations: NfL: neurofilament light chain.

Serum NfL was detectable in all investigated patients, including 11 with ATTRv and 60 with ATTRwt amyloidosis. The median serum NfL levels were significantly higher in ATTRv patients at 76 pg/ml (IQR: 47.9–116) compared to ATTRwt patients, with a median of 40 pg/ml (IQR: 26.5–52.5) (*p* = 0.011) (Fig. 1). Additionally, 55% (6/11) of ATTRv patients had serum NfL levels above the 95th age percentile, whereas only 10% (6/60) of ATTRwt patients did so.

### Survey of red flags symptoms

In addition to polyneuropathy, which was diagnosed in all patients with ATTRv (*n* = 11), the most common red flags in this cohort were autonomic dysfunction, affecting 73% (*n* = 8/11) of patients, along with GI complaints and cardiac involvement, each observed in 64% (*n* = 7/11) of patients. Interestingly, only 45% (*n* = 5/11) of patients with ATTRv reported a positive family history for polyneuropathy.

**Fig. 2:**
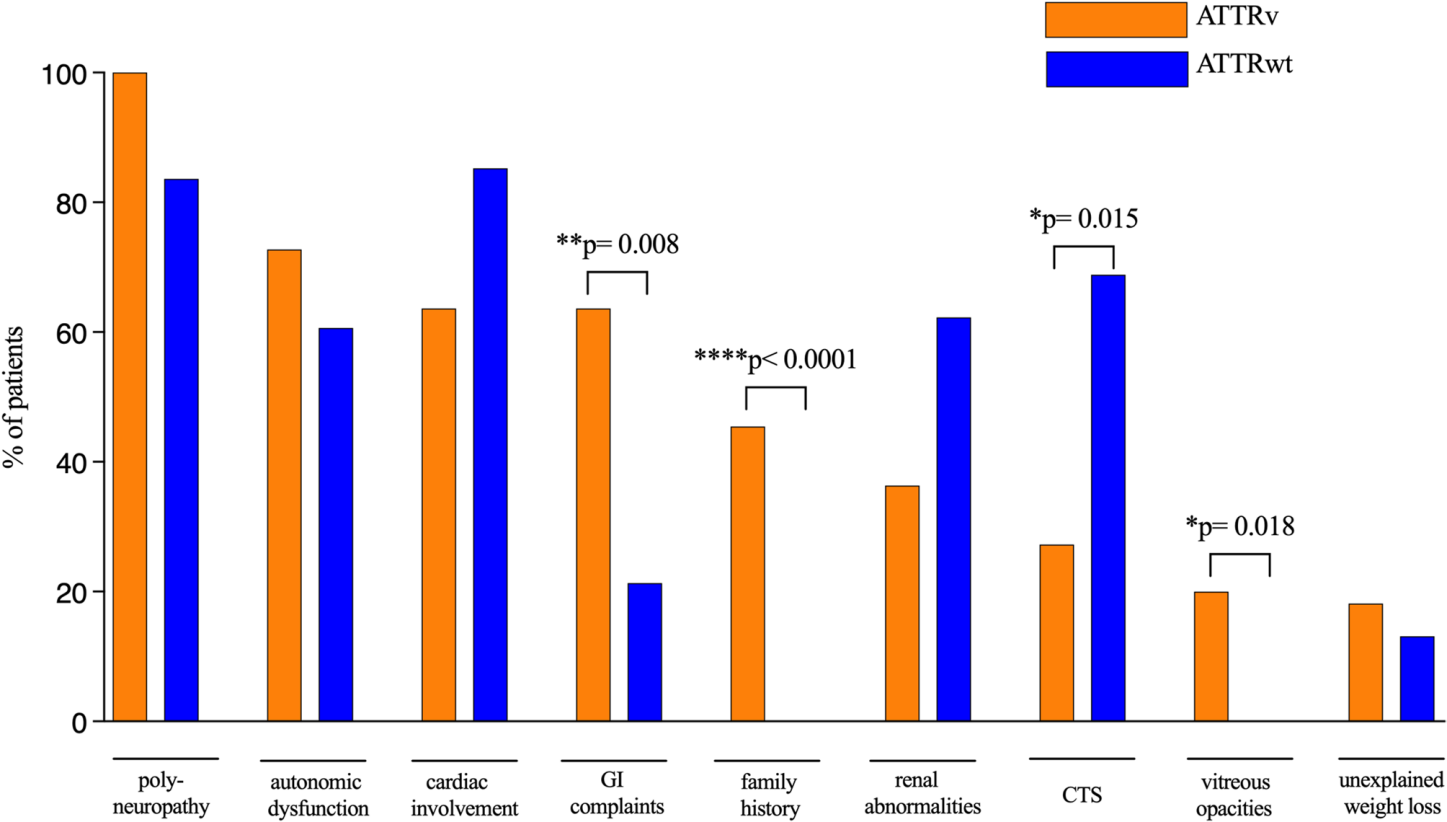
Comparison of red flag manifestations in ATTRv (*n* = 11) and ATTRwt (*n* = 61) patients. Note a wide overlap of the red flags between ATTRv and ATTRwt patients. A significant higher percentage of patients with ATTRv had gastrointestinal complaints and vitreous opacities, whereas CTS was more frequent in ATTRwt patients. Abbreviations: PNP: polyneuropathy; GI complaints: gastrointestinal complaints; CTS: carpal tunnel syndrome.

Remarkably, red flag symptoms were similarly frequent among patients with ATTRwt amyloidosis (*n* = 61). Polyneuropathy was observed in 84% (*n* = 51/61), cardiac involvement in 85% (*n* = 52/61), CTS in 69% (*n* = 42/61), and renal abnormalities in 62% of patients (*n* = 38/61). Notably, CTS occurred significantly more often in ATTRwt patients (*p* = 0.015) whereas ATTRv patients reported GI complaints more frequently (*p* = 0.008) and were more often prediagnosed with amyloid-associated vitreous opacities (*p* = 0.018). Taken together, all patients diagnosed with polyneuropathy showed at least one other red flag symptom (Fig. 2).

## Discussion

Our results highlight the fact that polyneuropathy is a common manifestation in patients with TTR amyloidosis, regardless of the genotype. There is a notable difference in the severity of polyneuropathy, including more pronounced subjective limitations, clinical disability, electrophysiological abnormalities, and higher serum NfL levels in ATTRv patients. However, the established red flag symptoms used to diagnose ATTRv amyloidosis were also commonly found in the majority of ATTRwt patients.

The neurological manifestations, particularly polyneuropathy, are widely recognized as key clinical features of ATTRv amyloidosis^27,28^. However, among all findings of ATTRwt amyloidosis, peripheral neuropathy has long been overlooked, since cardiomyopathy often dominates the clinical picture. Previous case reports and small case series suggested a high rate of polyneuropathy in ATTRwt patients^29,30^. In contrast, larger studies had reported a prevalence of peripheral neuropathy in ATTRwt ranging from 3% to 30.5%^3,30,31^. It is important to note that the larger studies often focus on highly selected patient populations, such as those initially diagnosed with ATTRwt cardiomyopathy, and therefore lacked detailed characterization of polyneuropathy in ATTRwt patients^30,31^.

Recently, Papagianni and colleagues performed detailed comparative investigations of both large and small fiber neuropathy in a mixed cohort of ATTRwt and ATTRv/asymptomatic gene carriers^11^. They reported large-fiber polyneuropathy in 43% of the ATTRv and 70% of the ATTRwt cohort and small-fiber impairment in all ATTRv and 80% of ATTRwt patients, which was considerably higher compared to previous reports^3^. However, the extended battery of diagnostic tests used in this study are only accessible to specialized neuromuscular centers and might not be widely available in the community setting. Another recently published study of 50 patients initially diagnosed with ATTRwt cardiomyopathy identified symmetric length-dependent polyneuropathy in 74% of the patients, underscoring the prevalence of polyneuropathy in this population. The polyneuropathy was primarily sensory, and autonomic symptoms were infrequent^10^.

In our investigation, we prospectively examined a large cohort of 72 individuals with ATTR amyloidosis during their initial interdisciplinary consultation at the Amyloidosis Center. We used the same diagnostic approach for both ATTRv and ATTRwt patients, allowing for direct comparison of the peripheral neuropathy findings between these two groups. The size difference of the two groups was attributed to the low prevalence of ATTRv in Germany^32^. Although Germany is a non-endemic area, the vast majority of ATTRv patients were affected by the p.*Val50Met* mutation (9/11). Due to the small cohort size in our study, different pathogenic variants were not adequately represented. Importantly, apart from referral to our specialized Amyloidosis Center, we have attempted to avoid the selection bias by all means. In addition to the high prevalence of polyneuropathy in the ATTRv group (100%), we also found a significantly high prevalence in ATTRwt patients (84%). Even after excluding patients with other risk factors for polyneuropathy, we still found 67% of ATTRwt patients with polyneuropathy. This represents a significantly higher prevalence of polyneuropathy compared to the age-matched general population, in which polyneuropathy is diagnosed in 12.6% of patients in the age group of 70 to 80 years^33^. The lack of skin biopsies represents a limitation of our study, as the prevalence of small fiber neuropathy remains unknown in our cohort, despite it being an early and frequent manifestation of ATTRv and ATTRwt^34–36^. Thus, the prevalence of polyneuropathy might still be underestimated in our study cohort. Although not designed to establish causality, our study suggests an association between ATTRwt and polyneuropathy based on its high frequency in the ATTRwt group. Despite the presence of typical symptoms and clinical findings, polyneuropathy was previously diagnosed in only 2% of ATTRwt patients, possibly due to confounding age-related factors like degenerative joint changes that can mask symptoms^37^. Our findings highlight the need for thorough history taking and clinical neurological examinations in ATTRwt patients, given that the majority of patients did in fact report typical symptoms, and even more showed objective clinical findings of polyneuropathy. This underscores the importance of early interdisciplinary investigations in patients presenting with peripheral neuropathy.

Through direct comparison of the polyneuropathy scores we found that the neurological deficits in ATTRwt patients were less severe compared to ATTRv patients. This was particularly notaceable in patients with advanced disease (e.g., FAP 3 and PND IV). This observation aligns with previous reports^11^. Consistent with the more severe clinical presentations in ATTRv patients, we observed more pronounced axonal damage in the ATTRv group on nerve conduction studies. While the severity of polyneuropathy in ATTRwt patients may not surpass that of ATTRv patients, it remains clinically significant. Even if polyneuropathy is restricted to only sensory domains, its impact on gait steadiness and fall risk cannot be overstated, particularly given the high prevalence of comorbidities such as lumbar spinal stenosis or degenerative joint disease in this aged ATTRwt population^37–39^.

Additionally, our study established serum filament light chain (NfL) as a marker for axonal injury in both forms of TTR amyloidosis, with its levels correlating with disease severity. Clinically and electrophysiologically more severely affected patients exhibited significantly higher NfL levels than less affected patients. While previous studies have established the utility of NfL as a marker for disease activity and severity in ATTRv amyloidosis^40–42^, data on its application in ATTRwt patients are scarce^43,44^. Our study demonstrated its utility in evaluation of both forms.

Red flag symptoms for early diagnosis of ATTR amyloidosis have previously been established for both ATTRv- and ATTRwt-associated disease subtypes. Polyneuropathy is a key finding for diagnosis of ATTRv amyloidosis. In comparison, cardiac abnormalities are required for diagnosis of ATTRwt diagnosis^8,45–47^. However, this classification may not reflect the clinical spectrum of both diseases^48,49^. In our study, all ATTRv patients exhibited at least one red flag symptom besides polyneuropathy. Interestingly, many red flags were also present in most ATTRwt patients, indicating systemic disease manifestations in both forms. This finding highlighted the overlapping clinical spectrum of ATTRv and ATTRwt amyloidosis. Still, significant differences were present, such as the prevalence of CTS in ATTRwt patients and more common GI complaints and vitreous opacities in ATTRv patients. The prevalence of renal abnormalities and autonomic dysfunction is difficult to estimate due to multiple confounding factors and the lack of biopsy^50^. For example, chronic kidney disease (CKD) is common in ATTRwt patients with cardiac involvement but may be confounded by age, heart failure, arterial hypertension, and diabetes mellitus status^51^. Similarly, the association of erectile dysfunction as a potential sign of autonomic dysfunction is confounded by age and cardiovascular disease status^52^.

## Conclusion

Our study from a large tertiary center in a non-endemic region demonstrates the high prevalence of polyneuropathy in patients with TTR amyloidosis, regardless of its genotype. Interestingly, the red flag manifestations commonly used to diagnose ATTRv amyloidosis were also prevalent in the majority of ATTRwt patients, making it challenging to differentiate between the two groups based solely on these indicators. However, a notable discrepancy in the severity of polyneuropathy was observed between ATTRv and ATTRwt patients, which may aid in the initial clinical diagnosis before a genetic diagnosis is established.

## Data Availability

Data are available from the corresponding author upon reasonable request.

## Abbreviations

AL amyloidosis: Systemic light chain amyloidosis
ANA: Antinuclear antibodies
ATTR amyloidosis: Transthyretin amyloidosis
ATTRv amyloidosis: Hereditary transthyretin amyloidosis
ATTRwt amyloidosis: Wild-type transthyretin amyloidosis
CKD: Chronic kidney disease
CMAP: Composite muscle action potential
CTS: Carpal tunnel syndrome
DGK: German Cardiac Society
DML: Distal motor latency
ENA: Extractable nuclear antigen antibodies
FAP: Familial amyloid polyneuropathy
FWL: F-wave latency
GI complaints: Gastrointestinal complaints
I-RODS: Inflammatory Rasch-built Overall Disability Scale
INCAT: Inflammatory Neuropathy Cause and Treatment disability score
IQR: Interquartile range
MCV: Motor conduction velocity
MRC Sum Score: Medical Research Council Sum Score
NCS: Nerve conduction studies
NfL: Neurofilament light chain
PND score: Polyneuropathy disability score
PNP: Polyneuropathy
SCV: Sensory conduction velocity
SD: Standard deviation
SNAP: Sensory nerve action potential
TTR: Transthyretin
